# Single-molecule FRET reveals the pre-initiation and initiation conformations of influenza virus promoter RNA

**DOI:** 10.1093/nar/gkw884

**Published:** 2016-09-30

**Authors:** Nicole C. Robb, Aartjan J. W. te Velthuis, Ralph Wieneke, Robert Tampé, Thorben Cordes, Ervin Fodor, Achillefs N. Kapanidis

**Affiliations:** 1 Biological Physics Research Group, Clarendon Laboratory, Department of Physics, University of Oxford, Parks Road, Oxford OX1 3PU, UK; 2 Sir William Dunn School of Pathology, University of Oxford, South Parks Road, Oxford OX1 3RE, UK; 3 Institute of Biochemistry, Biocenter, Goethe University Frankfurt, Max-von-Laue-Strasse 9, 60438 Frankfurt am Main, Germany; 4 Molecular Microscopy Research Group, Zernike Institute for Advanced Materials, University of Groningen, Nijenborgh 4, 9747 AG Groningen, The Netherlands

## Abstract

Influenza viruses have a segmented viral RNA (vRNA) genome, which is replicated by the viral RNA-dependent RNA polymerase (RNAP). Replication initiates on the vRNA 3′ terminus, producing a complementary RNA (cRNA) intermediate, which serves as a template for the synthesis of new vRNA. RNAP structures show the 3′ terminus of the vRNA template in a pre-initiation state, bound on the surface of the RNAP rather than in the active site; no information is available on 3′ cRNA binding. Here, we have used single-molecule Förster resonance energy transfer (smFRET) to probe the viral RNA conformations that occur during RNAP binding and initial replication. We show that even in the absence of nucleotides, the RNAP-bound 3′ termini of both vRNA and cRNA exist in two conformations, corresponding to the pre-initiation state and an initiation conformation in which the 3′ terminus of the viral RNA is in the RNAP active site. Nucleotide addition stabilises the 3′ vRNA in the active site and results in unwinding of the duplexed region of the promoter. Our data provide insights into the dynamic motions of RNA that occur during initial influenza replication and has implications for our understanding of the replication mechanisms of similar pathogenic viruses.

## INTRODUCTION

Negative-sense RNA viruses include a number of important human and animal pathogens; examples of disease caused by these viruses include measles, rabies, Ebola and influenza. Influenza A and B viruses are responsible for seasonal respiratory infections and influenza A viruses also cause occasional severe pandemics in humans resulting in substantial morbidity and mortality worldwide. Both type A and B viruses have eight viral RNA (vRNA) segments, while influenza C viruses have seven segments. The conserved 13 nucleotides at the 5′ end and 12 nucleotides at the 3′ of each vRNA segment are partially complementary and form a partly double-stranded promoter created by base-pairing between the two ends (1,2). This vRNA promoter is recognised and bound by the viral RNA polymerase (RNAP), which transcribes the vRNA genome into capped and polyadenylated mRNAs using short 5′-capped primers derived from host capped RNAs (3). The vRNA segments are also replicated by the RNAP in a primer-independent manner, via complementary RNA (cRNA) replicative intermediates, which in turn are used as templates to make more vRNA.

**Figure 1.**
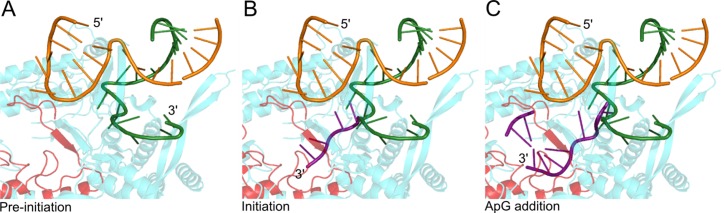
Structural models of influenza vRNA promoter conformations. (**A**) Structure of the pre-initiation state of the influenza virus vRNA. The RNAP (PDB code: 4WSB) is shown in blue, with the active site motifs highlighted in red, 5′ vRNA is orange and 3′ vRNA is green. (**B**) Model of the initiation state. The 3′ vRNA is reoriented into the active site; depicted by the template strand (purple) from the superposed Norwalk template–primer elongation complex (PDB code: 3BSO). (**C**) Model of replication initiation showing the position of the initial product (ApG) and translocation of the first two bases of the 3′ template through the active site, created by superimposition of the polio virus elongation complex RNA (PDB code 3OL7).

The influenza RNAP is a heterotrimeric complex comprising the PB1, PB2 and PA subunits. In recent years, crystal structures of influenza A, B and C RNAPs with bound 5′ and 3′ vRNA (4,5), 5′ cRNA (6) or no RNA (7), have emerged. These structures have revealed that the proximal 5′ and 3′ vRNA extremities (nucleotides 1–10 of the 5′ end and 1–9 of the 3′ end) are bound as predominantly single strands in distinct binding sites on the protein, with the distal part of the vRNA promoter forming a base-paired duplex region (nucleotides 11–14 of the 5′ end and 10–13 of the 3′ end) that projects away from the body of the RNAP (4,5). The 5′ extremity is bound in a deep pocket, clearly visible in all the crystal structures with RNA, while the 3′ extremity is usually not resolved, suggesting that it is flexible and can occupy multiple positions. The single-stranded 3′ extremity of the vRNA template is only resolved in one of the available RNAP structures (5), which shows that the 3′ RNA within this structure does not enter the RNAP active site but is bound in an alternative location on the surface; this conformation is designated the ‘pre-initiation’ state (Figure 1A). The sequence-specific nature of the 3′ end binding in the pre-initiation state and conservation of interacting residues is suggestive of a functionally important binding site, implying that a mechanism for relocating the 3′ end into the RNAP active site during initiation of RNA synthesis must exist. A model of the RNAP in an ‘initiation state’, in which the 3′ terminus of the vRNA template is found in the active site, can be created using RNA from the Norwalk virus RNAP initiation complex (Figure 1B) (5). Similarly, superposition of RNA from the poliovirus elongation complex with the influenza RNAP allows the construction of a model for initial replication showing the positions of the first two incoming nucleotides and translocation of the first two bases of the 3′ template through the active site (Figure 1C) (5). Currently, it is unclear how the transition of the RNAP from a pre-initiation to initiation state is regulated.

The 3′ and 5′ termini of the cRNA promoter are predicted to form a secondary structure similar to that of the vRNA promoter (8,9), and a crystal structure of the RNAP with a short 5′ cRNA terminus shows that the conformation of the first twelve residues of the 5′ cRNA is virtually identical to that of the vRNA 5′ terminus (6). This suggests that the 3′ end of the cRNA promoter also occupies the same site as the 3′ end of the vRNA template on the surface of the RNAP, and a transition from ‘pre-initiation’ to ‘initiation’ mode may also occur on the cRNA template; however, no information on the binding of the 3′ cRNA terminus is currently available.

In this work, we set out to explore the dynamic motions of the influenza viral RNA during RNAP binding and replication initiation. We built upon a previous study from our group that used a sensitive, solution-based, single-molecule Förster resonance energy transfer (smFRET) assay to measure distances between fluorescent dyes located on the viral RNA promoter upon RNAP binding (10); this assay has the advantage over ensemble experiments that it allows heterogeneous and dynamic populations to be distinguished and characterized. We show that the vRNA promoter exhibits two different conformations upon RNAP binding, corresponding to pre-initiation and initiation states. Addition of an ApG dinucleotide mimicking the replication initiation product affects the conformational equilibrium and stabilises the 3′ vRNA terminus in the active site. We see similar conformations for the cRNA, suggesting a functional importance of the pre-initiation state for both promoters. Dinucleotide addition destabilises the duplex region of the promoter vRNA, suggesting that this region is unwound early in replication. The similar architecture and RNA-binding modes of the RNAPs of all segmented negative-strand viruses suggests a common evolutionary origin and mechanism of RNA synthesis. The results from this work are therefore applicable for understanding the mechanisms of promoter binding and replication initiation of similar pathogenic viruses.

## MATERIALS AND METHODS

### RNA

RNA corresponding to the 3′ and 5′ conserved ends of the neuraminidase vRNA or cRNA gene segment were custom synthesized by IBA (Gottingen, Germany) and were labelled with the fluorophores Cy3 or Cy3B (GE Healthcare) and ATTO647N (Sigma) as previously described (11), before being purified by gel electrophoresis. RNAs were annealed in hybridization buffer (50 mM Tris-HCl pH 8.0, 1 mM EDTA, 500 mM NaCl). RNA sequences are described in the text and figures.

### Protein

Recombinant A/NorthernTerritories/60/68 (H3N2) viral RNAP with a protein-A tag on the PB2 subunit was expressed using the Multibac system (12) in Sf9 insect cells and affinity purified using IgG-sepharose followed by size exclusion chromatography as previously described (10,13). Recombinant A/WSN/33 (H1N1) histidine-tagged RNAP (with a his-10 tag inserted at residue 200 on the PB1 subunit and a protein-A tag on the C-terminus of the PB2 subunit) was expressed in mammalian cells and purified using IgG-sepharose as previously described (14).

### Single-molecule fluorescence spectroscopy

A custom-built confocal microscope was used for single-molecule FRET experiments as previously described (15–17) and the setup was modified to allow alternating-laser excitation of donor and acceptor fluorophores (18,19). RNAP at a final concentration of 100 or 200 nM was incubated with fluorescently labelled RNA at a final concentration of 1 nM for 15 min at 28°C in buffer A (50 mM Tris–HCl (pH8), 500 mM NaCl, 10 mM MgCl_2_, 100 μg/μl BSA, 1 mM DTT and 5% glycerol) before being diluted into buffer B (50 mM Tris–HCl (pH8), 100 mM KCl, 10 mM MgCl_2_, 100 μg/μl BSA, 1 mM DTT and 5% glycerol) to a final RNA concentration of 100 pM for confocal analysis. For RNAP labelling, 2–3 nM of Cy3- or ATTO647N-labelled *tris*NTA was incubated with 100 nM RNAP and 1 nM fluorescently labelled RNA in buffer A for 15 min at 28°C, before being diluted into buffer B for confocal analysis. For nucleotide addition, 500 μM ApG was incubated with 100 nM RNAP and 1 nM fluorescently labelled RNA in buffer A for 15 min at 28°C, before being diluted into buffer B for confocal analysis. The average excitation intensities were 250 μW at 532 nm and 60 μW at 635 nm. Custom-written LabVIEW software was used to register and evaluate the detected signal.

### Data analysis

Fluorescence photons were assigned to either donor or acceptor-based excitation with respect to their photon arrival time and two characteristic ratios, the fluorophore stoichiometry S and apparent FRET efficiency E*, were calculated for each fluorescent burst above a certain threshold, yielding a two-dimensional histogram (11,18,19). One-dimensional E* distributions for donor-acceptor species were obtained by using a 0.4 < *S* < 0.8 threshold. These E* distributions could be fitted using a Gaussian function, yielding the mean E* value for a certain distribution and an associated standard deviation.

### Modelling of the influenza virus RNA and FRET positioning and screening software (FPS)

A model which incorporated the influenza A polymerase (4) and residues 1–14 of the 5′ RNA and 1–13 of the 3′ RNA from the fluB2 structure (5) was created in PyMol, and the duplex RNA was extended to positions 18 for the 5′ and 17 for the 3′ RNA using the DuplexFold module of the RNAstructure webserver (20) and the Chimera plugin Assemble2 (21). The attachment point for each dye was identified (a uridine on the C5′ of the sugar residue). The Cy3 dye was characterized by a linker length of 14.2 Å, a linker width of 4.5 Å and dye radii of 8.2, 3.3 and 2.2 Å (*x, y* and *z*, respectively) (10). The ATTO647N dye was characterized by a linker length of 17.8 Å, a linker width of 4.5 Å and dye radii of 7.4, 4.8 and 2.6 Å. FRET positioning and screening software (FPS) was used to calculate the accessible volumes of the dyes and the average dye positions (represented as spheres in the figures) (22,23). Distances between the average positions of the FRET dye pairs were determined using the ‘measure distance’ function available in PyMol.

### *In vitro* transcription assays

Transcription was carried out using double-stranded RNA templates in the presence of ApG as a primer, as previously described (24). Sequences of the RNA templates used are described in the figure legend. Reactions were incubated for 2 h at 30°C before being stopped by addition of loading dye (90% formamide, 10 mM EDTA, bromophenol blue, and xylene cyanol), analysed on a 6 M urea, 20% polyacrylamide sequencing gel and visualized by autoradiography.

## RESULTS

### Polymerase-bound vRNA exhibits multiple structural conformations

**Figure 2.**
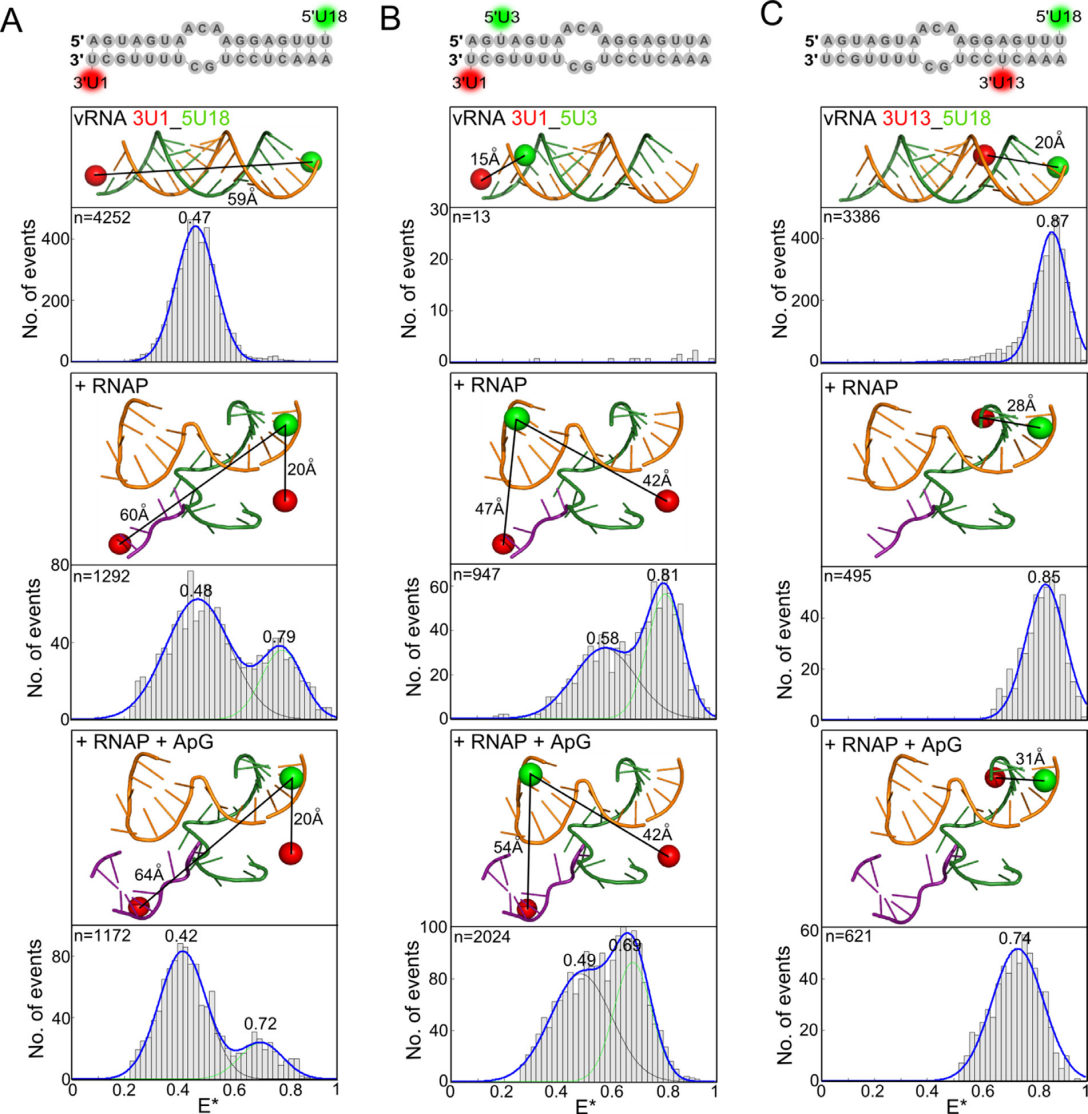
Influenza virus RNAP-bound vRNA promoter adopts multiple conformations. (**A**) Annealed RNAs, corresponding to the 3′ (3′-UCGUUUUCGUCCUCAAA) and 5′ (5′-AGUAGAAACAAGGAGUUU) ends of vRNA, labelled with donor and acceptor fluorophores at positions 1 on the 3′ end and 18 on the 5′ end (**A**), 1 on the 3′ end and 3 on the 5′ end (**B**) or 13 on the 3′ end and 18 on the 5′ end (**C**), were analysed alone (top panels), in the presence of 100 nM RNAP (middle panels), or in the presence of 100 nM RNAP and 500 μM ApG (lower panels) by single-molecule FRET spectroscopy of diffusing molecules. Histograms from three independent experiments were merged. Ratio E* represents the uncorrected FRET efficiency, n represents the number of molecules and curves were fitted with Gaussian functions to determine the centre of the distributions.

To examine the conformations of viral RNA within RNAP–vRNA complexes, we incubated purified recombinant RNAP from influenza A/NorthernTerritories/60/68 (H3N2) (Supplementary Figure S1A) with short, partially complementary RNAs corresponding to the conserved 5′ and 3′ termini of the vRNA, fluorescently labelled with a donor dye at position 18 on the 5′ strand and an acceptor dye at position 1 on the 3′ strand (Figure 2A) (10,13). We demonstrated that the fluorescent dyes had no effect on the ability of the RNAP to extend ApG using an *in vitro* replication assay (Supplementary Figure S1B). Single-molecue FRET was measured on complexes diffusing in solution, and apparent FRET efficiency values (E*) reporting on the donor-acceptor distance between the fluorophores on the RNA were depicted as merged histograms from three independent experiments and fitted with Gaussian functions to determine the mean FRET efficiency of the distributions (17,25).

In the absence of RNAP, the 5′ and 3′ termini of vRNA form a partially double-stranded structure, which produced a single FRET population with a mean E* of 0.47 (Figure 2A, top). FRET positioning and screening software (FPS) (22,23) was used to calculate the accessible volumes of the dyes (taking into account their size, linker lengths and constraints by the protein if present) and an estimated donor-acceptor distance of 59 Å was calculated using the mean positions of the dyes (depicted as spheres in the figures; Figure 2A, top). The addition of RNAP (at a final concentration of 100 nM) resulted in a bimodal FRET distribution with mean E* values of 0.48 and 0.79 (Figure 2A, middle). We modelled the 3′ dye at position 1 on either the vRNA pre-initiation structure (5), or on the superposed Norwalk virus RNA in which the 3′ terminus goes into the active site (Figure 2A, middle). A distance of 20 Å was measured when the 3′ dye was in the pre-initiation conformation on the surface of the protein, while a distance of 60 Å was measured between dyes at 5U18 and 3U1 in the active site of the RNAP. These distances correlate with the observed bimodal FRET distribution; with the high-FRET population corresponding to the shorter distance of 20 Å and the low-FRET population corresponding to the greater distance of 60 Å, similar to that of dsRNA only (59 Å). We therefore hypothesized that the high-FRET population corresponds to the RNA in the pre-initiation state, while the low-FRET population corresponds to the vRNA promoter in the initiation state (i.e., in the active site of the RNAP).

The addition of ApG, mimicking the product of *de novo* initiation, also resulted in a bimodal FRET distribution, centered at E* values of 0.42 and 0.72 (Figure 2A, bottom). FPS modelling of the dye at 3U1 using RNA from the superposed poliovirus elongation complex showed that the high-FRET population is likely to reflect RNA in the pre-initiation state (a short distance of 20 Å), while the low-FRET population reflects vRNA promoters in which the template RNA has started to translocate through the active site (a longer distance of 64 Å). The shift of the low-FRET population to an E* value of 0.42, compared to that measured when no ApG is present (E* ∼ 0.48), can be explained by the increased distance of the dye at 3U1 to the dye at 5U18 as the template RNA moves through the active site, while the shift of the high-FRET population to a lower mean E* value (from 0.79 to 0.72) may reflect movement of the 5U18 dye during initial replication. ApG addition resulted in an increase in the low-FRET population (from 58% to 78% of the total number of FRET events) and a decrease in the high-FRET population (from 42% to 22%) compared to when no ApG was present (Figure 2A, bottom), suggesting that the presence of a dinucleotide stabilizes the 3′ end of the vRNA in the active site.

The FPS modelling showed that the accessible volume of the acceptor ATTO647N dye at 3U1 in the RNAP active site was much more restricted than that of the donor Cy3 dye at position 5U18 or the ATTO647N dye at position 3U1 in the pre-initiation state (Supplementary Figure S2A). In order to confirm that the changes in FRET efficiency that we observed were due to changes in distance, rather than restricted orientation of the dyes, we used an RNA construct where the dyes were swapped so that a Cy3 dye was placed at position 3U1 and an ATTO647N dye was placed at position 5U18. The accessible volume modelling of the dyes showed that the Cy3 dye in the RNAP active site was constrained in a very different way to the ATTO647N dye (Supplementary Figure S2B), however we observed a similar result to that shown in Figure 2A; RNA only gave a single FRET population whilst upon RNAP addition two distinct conformations were observed (Supplementary Figure S2C). This strongly suggests that our observations reflect changes in distance rather than depending on limited rotation of the fluorophores, as it is highly unlikely that the rotational freedom of different fluorophores, with different linker lengths, would be constrained in exactly the same way.

To test the hypothesis of the existence of two RNAP-bound vRNA promoter conformations, we used a labelling strategy where a donor dye was placed at position 3 on the 5′ end, and an acceptor dye at position 1 on the 3′ vRNA (Figure 2B). In this configuration, the two fluorophores are in such close proximity to each other (a distance of less than ∼20 Å) that they undergo contact-induced quenching, which suppresses fluorescence emission (10,11,17,26). Fluorescence can be measured, however, if the two termini separate upon RNAP binding. As expected, no FRET was observed for the unbound vRNA (Figure 2B, top); however, when RNAP was added, a bimodal FRET distribution was observed (with mean E* values of 0.58 and 0.81) (Figure 2B, middle). Given that the RNA alone does not give a fluorescent signal, both low-FRET and high-FRET distributions must correspond to RNAP-bound RNA species. We note that additional heterogeneity in the RNA structure may exist that is not represented in our models (especially since there is only one available structural model for the 3′ RNA in a pre-initiation complex); however, the use of FPS modelling suggested that the low-FRET population corresponds to the initiation complex (a distance of 47 Å), while the high-FRET population likely represents the pre-initiation complex (a shorter distance of 42 Å). Addition of ApG again resulted in an increase in the low-FRET population (from 41% to 50%) and decrease in the high-FRET population (from 59% to 50%) (Figure 2B, bottom). We note that there is a larger decrease in the high-FRET population for 3U1_5U18 compared to 3U1_5U3 upon ApG addition; it is possible that in the 3U1_5U18 construct some fluorescence quenching of the dyes occurs when they are in close proximity in the pre-initiation state, resulting in an under-estimation of the high-FRET state. Overall, however, evidence from both constructs supports the notion that the presence of a product mimicking initial replication stabilises the 3′ end of the vRNA in the RNAP active site.

To gain further insight into the conformational changes of the vRNA promoter upon RNAP binding and replication initiation, we measured FRET between dyes at position 18 on the 5′ RNA and 13 on the 3′ RNA, both located in the distal region of the vRNA promoter. The dyes are close together in this configuration, generating a high-FRET signal in the unbound state (Figure 2C, top). When RNAP was added, only a single FRET distribution was observed (centred at E* ∼ 0.85) (Figure 2C, middle), consistent with the conformation of the distal part of the vRNA promoter remaining the same, independent of whether the 3′ end is located in its binding site at the surface of the RNAP or in the active site. Addition of ApG shifted the FRET distribution to lower values (centred at E* ∼ 0.74), suggesting an increase in the distance between the two dyes (Figure 2C, bottom), consistent with FPS modelling in which the dye at 3U13 moves forward by two base-pairs as the RNA template translocates through the active site. This conformational change in the double-stranded distal part of the vRNA promoter may correspond to the early stages of the separation of the 3′ and 5′ termini to allow the unhindered movement of the vRNA template through the RNAP active site.

**Figure 3.**
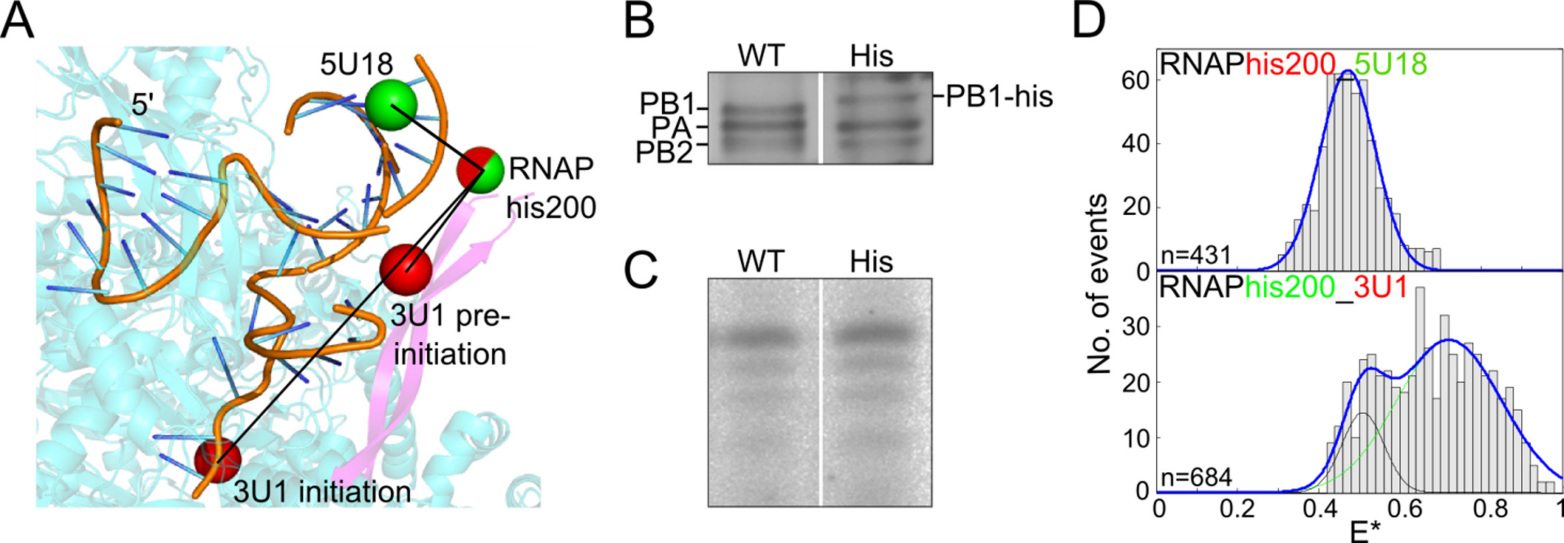
Fluorescent RNAP reveals multiple conformations of the 3′ terminus of the vRNA promoter. (**A**) Model of the influenza vRNA promoter and polymerase showing average dye positions. The 3′ vRNA is in orange and shows both the pre-initiation and initiation states, the polymerase is shown in blue, with the PB1 ß-ribbon highlighted in purple. A deca-histidine tag was inserted after residue 200 in the PB1 subunit (the additional histidine residues are not depicted in the figure; the red/green sphere represents the dye modelled on residue 200), while fluorescent dyes on the RNA are shown as green or red spheres. (**B**) Silver-stained polyacrylamide gel of wildtype (WT) and histidine-tagged (His) RNAP. (**C**) *In vitro* transcription assay showing that the histidine tag does not interfere with polymerase activity. (**D**) ATTO647N-labelled histidine-tagged polymerase (RNAPhis200) and vRNA promoter, labelled with a Cy3 dye at position 18 on the 5′ end, were analysed by single-molecule FRET spectroscopy of diffusing molecules (upper panel). Cy3-labelled histidine-tagged polymerase (RNAPhis200) and vRNA promoter, labelled with an ATTO647N dye at position 1 on the 3′ end, were analysed in a similar fashion (lower panel).

To provide further support for the existence of the 3′ vRNA terminus in multiple structural conformations upon RNAP binding, we measured FRET between a fluorescent dye on the RNA and one placed on the RNAP. We chose a labelling approach in which fluorophore-conjugated *tris-*nitrilotriacetic acid (*tris*NTA) (27–30) was bound to an internal His_10_ tag placed at amino acid 200 within a ß-ribbon of the PB1 subunit of the RNAP (PB1 his200). Labelling was carried out in situ, resulting in a large background from unbound dye-conjugated *tris*NTA (Supplementary Figure S3), however a clear FRET signal could be observed. FPS modelling of the dye positions provided an estimation of the number of FRET populations to expect from each labelling scheme; however, accurate donor-acceptor distance calculations were not possible due to the additional histidine residues not being present in the structure (Figure 3A). The His_10_ tag had no effect on RNAP trimer formation (Figure 3B) or *in vitro* activity (Figure 3C). When we used the labelled RNAP in an assay in which the dye on the vRNA was placed at position 18 on the 5′ end (a position insensitive to the 3′ RNA conformation), a single FRET distribution was observed (Figure 3D, top panel). Significant movement of the ß-ribbon of the RNAP can be excluded by the narrow FRET distribution (standard deviation 0.07). When the dye was moved to position 1 on the 3′ vRNA (a position which should give different FRET values depending on whether the 3′ template is in the pre-initiation configuration or in the active site), we indeed observed two FRET distributions (Figure 3D, lower panel). Together, these observations are consistent with our conclusion that the 3′ terminus of the vRNA exhibits two different conformations upon RNAP binding, corresponding to pre-initiation and initiation states.

### Polymerase-bound cRNA also exhibits multiple structural conformations

A recent crystal structure of the influenza RNAP (6) showed that the extreme terminus of the 5′ cRNA adopts a stem–loop structure virtually identical to that of the 5′ vRNA (Supplementary Figure S4), however the structure and binding site of the 3′ cRNA terminus is currently unknown. Prior to investigating the structural conformations of the cRNA promoter, we characterised the binding affinity of the RNAP for either single-stranded or double-stranded cRNA using fluorescent anisotropy or smFRET (10). Experiments with the 5′ cRNA terminus, 3′ cRNA terminus and the cRNA promoter yielded K_d_ values of 44 nM, >1 μM and 13 nM, respectively (Supplementary Figure S5). Similar experiments carried out previously with vRNA showed that the *K*_d_ of the RNAP for the 5′ vRNA terminus, the 3′ vRNA terminus, and the vRNA promoter was 2.2 nM, >1 μM and 0.4 nM, respectively (10). The RNAP therefore has significantly lower binding affinity for the cRNA promoter than the vRNA promoter.

**Figure 4.**
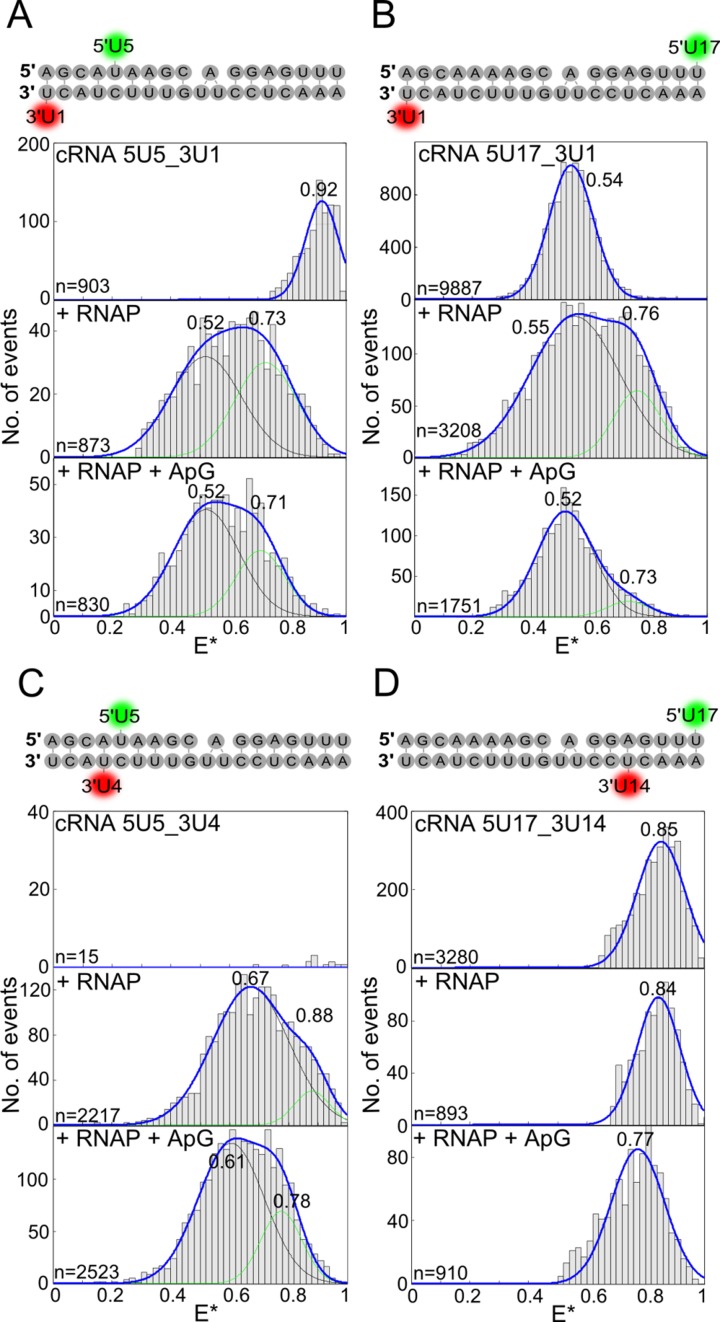
Influenza virus RNAP-bound cRNA promoter adopts multiple conformations. (**A–D**) cRNA promoters (sequences and labelling positions depicted in the figure) were labelled with donor and acceptor fluorophores and either analysed alone (top panels), or incubated with a final concentration of 200 nM RNAP (middle panels), or 200 nM RNAP and 500 μM ApG (lower panels) before single-molecule FRET spectroscopy on diffusing molecules was carried out. Histograms from three independent experiments were merged. Ratio E* represents the uncorrected FRET efficiency, *n* represents the number of molecules and curves were fitted with Gaussian functions to determine the centre of the distributions.

To gain insight into the conformation of the cRNA 3′ end upon RNAP binding, we labelled a cRNA promoter at position 5 on the 5′ end and 1 on the 3′ end; labelled promoters were active in an ApG extension assay (Supplementary Figure S6). The promoter alone gave a single high-FRET population with a mean E* of 0.92 (Figure 4A, top panel). Addition of RNAP to a final concentration of 100 nM resulted in a bimodal FRET distribution; however, some high-FRET population still remained (Supplementary Figure S7). Increasing the final RNAP concentration to 200 nM abolished the high FRET population, while leaving the bimodal FRET distribution centred at an E* of 0.52 and 0.73 (Figure 4A, middle panel). Thus, a higher concentration of RNAP was required to achieve full binding of the cRNA promoter compared to the vRNA promoter, in agreement with our conclusion that the binding affinity of the RNAP for the cRNA promoter is lower than that for the vRNA promoter.

The presence of a bimodal FRET distribution using the 5U5 and 3U1 labelling strategy showed that the cRNA 3′ end adopts two different conformations when bound by RNAP, possibly corresponding to pre-initiation and initiation states similar to that of the vRNA promoter. ApG addition resulted in a decrease in the high-FRET population (Figure 4A, bottom panel). Addition of RNAP resulted in a similar bimodal distribution and a decrease in the high-FRET population in the presence of ApG when the 5′ dye was moved to position 17 (Figure 4B). An RNA in which the dye pair was placed at position 4 on the 5′ end and position 1 on the 3′ end, which ensured that their fluorescence was quenched in the absence of RNAP (Figure 4C, top panel), also resulted in two FRET populations (Figure 4C, middle and bottom panels), confirming that both high-FRET and low-FRET populations represent RNAP-bound RNA. A cRNA promoter with dyes at position 17 on the 5′ end and 14 on the 3′ end, which is insensitive to conformational changes of the extreme 3′ end, produced only a single FRET distribution when RNAP was added, in agreement with the observations on the vRNA promoter (Figure 4D). Although no modelling of the dye positions is possible due to the lack of structural information for the cRNA 3′ end, these four labelling schemes confirm that the extreme cRNA 3′ terminus is also able to adopt two different conformations upon RNAP binding; the similarity of these data to that of the vRNA promoter suggests that these two conformations may correspond to pre-initiation and initiation states. Whilst interpretation of the effect of dinucleotide addition is more complicated for cRNA than vRNA, we can see a change in the ratio of the two FRET populations upon dinucleotide addition, and for those labelling schemes that are analogous to that of the vRNA template the change in the FRET populations follows a similar trend.

### The double-stranded distal region of the vRNA promoter is destabilised during initial replication

**Figure 5.**
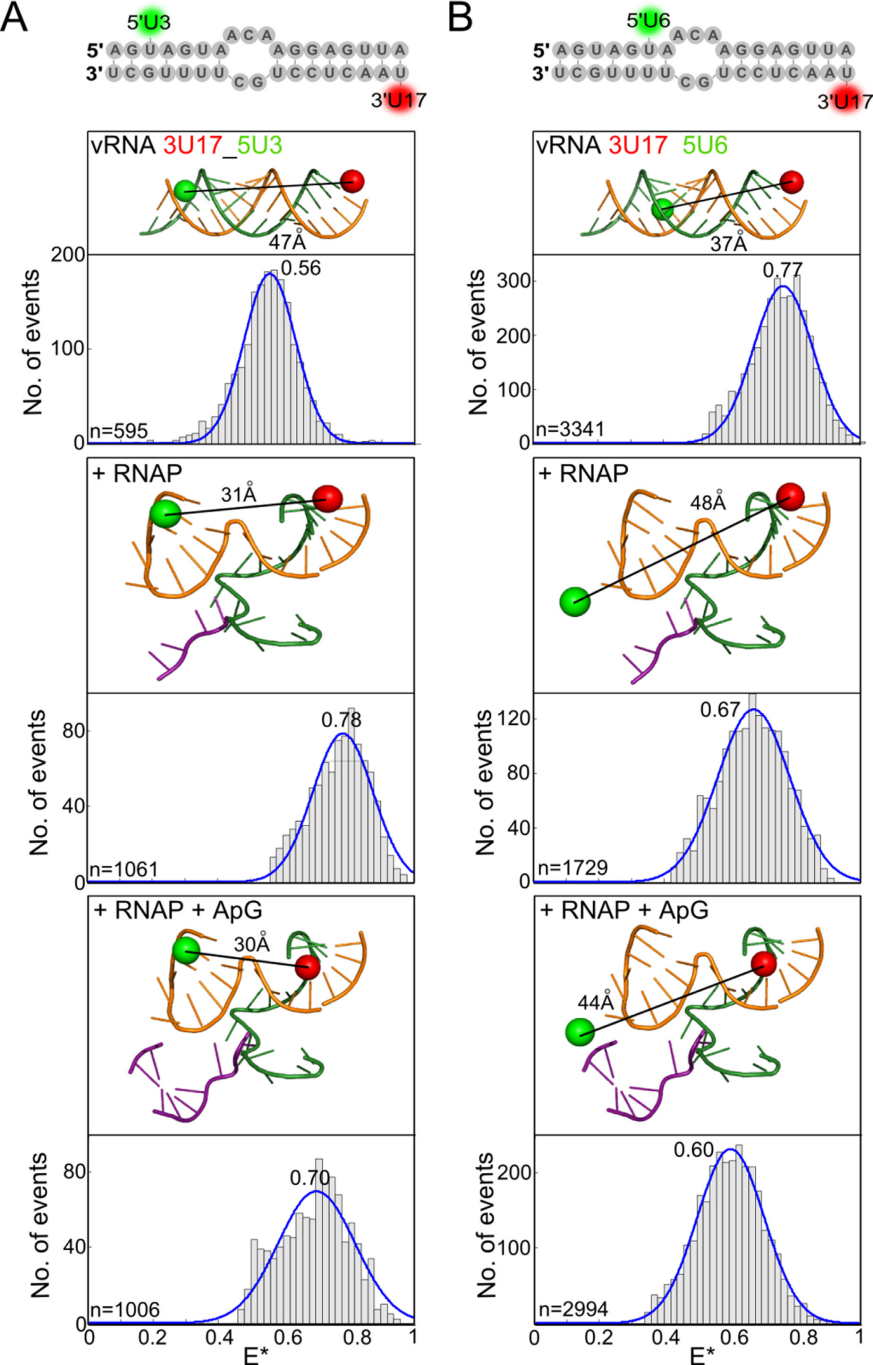
The duplex region of the vRNA promoter is destabilised during initial replication. Annealed RNAs, corresponding to the 5′ (5′-AGUAGAAACAAGGAGUUU) and 3′ (3′-UCGUUUUCGUCCUCAAA) ends of the vRNA promoter, were labelled with donor and acceptor fluorophores at positions 17 on the 3′ end and 3 on the 5′ end (**A**), or positions 17 on the 3′ end and 6 on the 5′ end (**B**), and were either analysed alone (top panels), or incubated with a final concentration of 100 nM RNAP (middle panels), or 100 nM RNAP and 500 μM ApG (lower panels) before single-molecule FRET spectroscopy on diffusing molecules was carried out. Histograms from three independent experiments were merged. Ratio E* represents the uncorrected FRET efficiency, *n* represents the number of molecules and curves were fitted with Gaussian functions to determine the centre of the distributions.

Having investigated changes in conformation of the proximal region of the vRNA promoter during RNAP binding and dinucleotide addition, we searched for structural changes that occur in the double-stranded distal region of the promoter (nucleotides 11–18 on the 5′ strand and 10–17 on the 3′ strand in our constructs). The 5′ and 3′ termini in this region must separate during processive elongation, when the 3′ end of the template vRNA translocates through the active site while being copied into nascent cRNA. However, it is unclear at which point the two termini separate. To detect changes in the distal region, we first placed a dye pair at position 3 on the 5′ vRNA and position 17 on the 3′ vRNA. This resulted in a single FRET distribution centred at an E* value of 0.56 for the RNA only, and a single FRET distribution centred at an E* of 0.78 when RNAP was added (Figure 5A, top and middle). In agreement with FPS modelling of the dye positions, the location of the 3′ terminus of the vRNA at the surface binding site or in the RNAP active site cannot be distinguished with a dye at 3U17. Modelling suggested that ApG addition should result in the forward translocation of the 3′ end of the vRNA by up to two nucleotides in the RNAP active site, thus bringing the dye at 3U17 closer towards the dye at 5U3 and increasing the FRET efficiency between them. Interestingly, we observed a decrease in FRET efficiency (from E* ∼ 0.78 to 0.70) upon ApG addition, consistent with the dyes moving further apart, suggesting that dinucleotide addition destabilised the distal region (Figure 5A, bottom). Similar results were observed when the 5′ dye was moved to position 6 (Figure 5B). Measurements taken at 37°C, rather than at room temperature, also produced similar results, confirming that the duplex in unbound RNA is stable at a higher temperature (Supplementary Figure S8).

**Figure 6.**
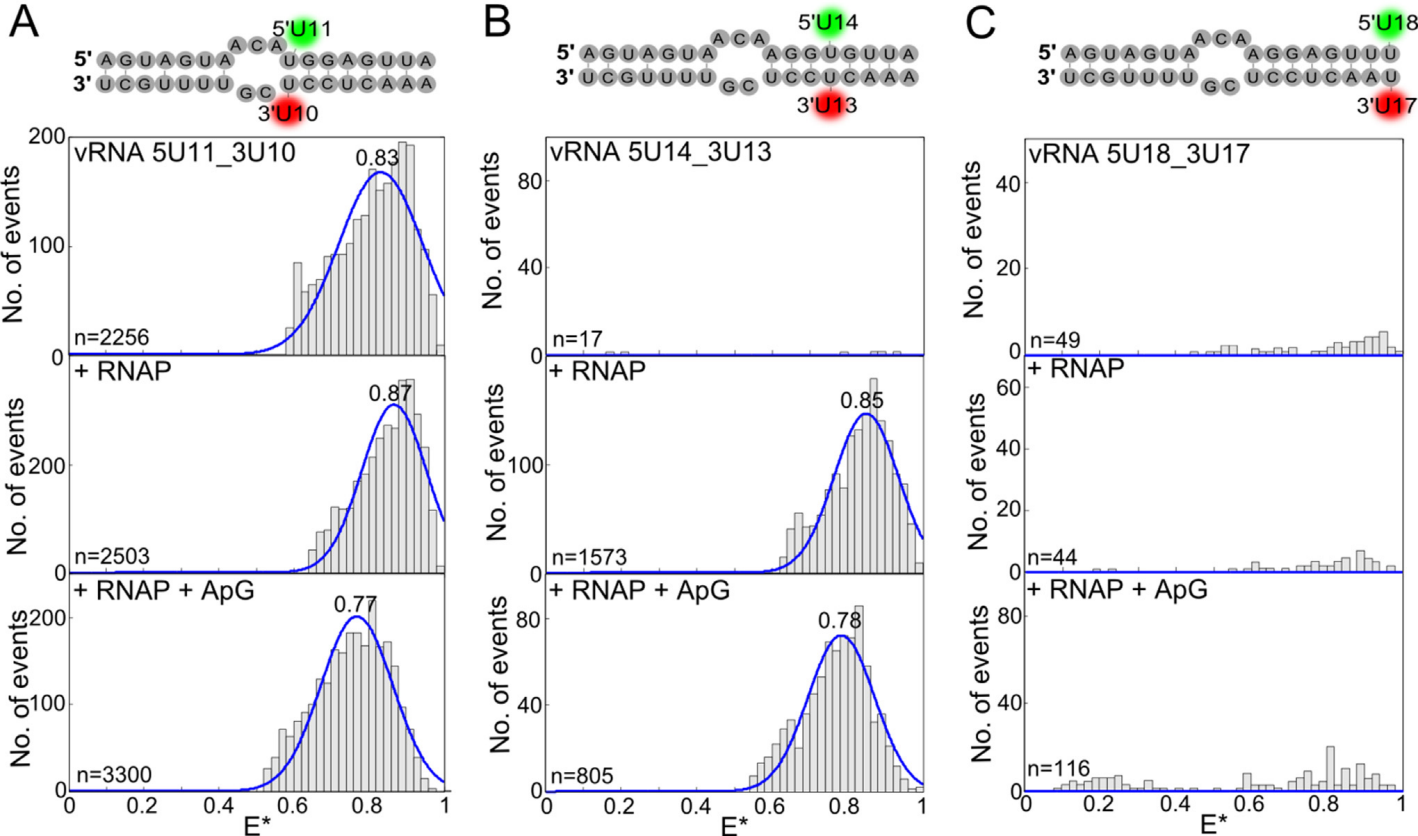
Quenchable FRET shows that the duplex region of the vRNA promoter is unwound in a processive manner. Annealed RNAs, corresponding to the 5′ and 3′ ends of the vRNA promoter (sequences depicted in the figure), were labelled with donor and acceptor fluorophores at positions 10 on the 3′ end and 11 on the 5′ end (**A**), positions 13 on the 3′ end and 14 on the 5′ end (**B**) or positions 17 on the 3′ end and 18 on the 5′ end (**C**) were either analysed alone (top panels), or incubated with a final concentration of 100 nM polymerase (middle panels), or 100 nM polymerase and 500 μM ApG (lower panels) before single-molecule FRET spectroscopy on diffusing molecules was carried out. Histograms from three independent experiments were merged. Ratio E* represents the uncorrected FRET efficiency, *n* represents the number of molecules and curves were fitted with Gaussian functions to determine the centre of the distributions.

In order to investigate the destabilisation of the distal region of the vRNA promoter further we used our quenchable FRET assay to detect separation of the RNA strands. We initially placed the dyes opposite each other, at positions 11 on the 5′ vRNA and 10 on the 3′ vRNA, which unexpectedly resulted in a high-FRET distribution for the unbound RNA (E* ∼ 0.83), rather than the fluorescence quenching that was expected (Figure 6A, top). Addition of RNAP did not alter the FRET distribution significantly (E* ∼ 0.87) (Figure 6A, middle). The dyes on the unbound RNA must be sufficiently separated to allow FRET to occur, suggesting transient opening and closing of the double-stranded RNA in this region. It is possible that this flexibility occurs because residues 10 and 11 follow directly after a short unpaired region in the promoter, or alternatively because mutation of the adenosine at position 11 to a uridine, necessary for labelling purposes, abolishes the base-pairing with the uridine at position 10. The mutation at position 11 did not affect the ability of the RNAP to extend ApG; however, placing a dye at position 10 on the 3′ RNA resulted in the loss of short, but not full-length, replication products (Supplementary Figure S1B). The presence of the dye could also therefore contribute towards the flexibility of the RNA in this region. Nevertheless, addition of ApG shifted the population to lower FRET (E* ∼ 0.77), consistent with the dyes moving further apart, indicating that dinucleotide addition can act to destabilise this early distal region (Figure 6A, bottom).

Similar experiments with the dye pair located in the middle of the duplex region, at positions 13 and 14 on the vRNA, gave no FRET for the dsRNA only, as expected for fully double stranded RNA, while addition of RNAP resulted in a FRET distribution centred at E* ∼ 0.85 (Figure 6B, top and middle). Again, ApG addition resulted in a shift of the mean E* value to lower FRET (E* ∼ 0.78) and was hence sufficient to destabilise this region (Figure 6B, bottom). Interestingly, when the dye pair was moved to the far end of the duplex, at positions 17 and 18, no FRET was observed when either RNAP or ApG was added (Figure 6C). This suggests that although the far end of the duplex region may be destabilised during initial replication the strands are not sufficiently separated to allow FRET to occur; in the absence of a crystal structure of a replicating RNAP it is difficult to predict how far apart the two RNA strands will actually move. Taken together, however, our data suggests that during initial de novo replication the duplex region is unwound in a processive manner (in a downstream direction) rather than being destabilised completely as a whole.

## DISCUSSION

The main breakthrough of our study was to obtain information on the dynamic motions of the viral RNA occurring within the influenza RNAP/RNA complex that cannot be directly observed in crystallographic structures. Intriguingly, to date none of the RNAP structures show the 3′ terminus of the vRNA template in the active site; instead, it is bound in an inactive conformation on the exterior of the protein (5), designated the ‘pre-initiation’ state in our studies. Our single-molecule measurements of RNAP-bound vRNA labelled at the extreme 3′ terminus revealed a low-FRET and high-FRET population in the absence of nucleotides. Similar results were observed in a previous publication of ours, where the low-FRET population was attributed to unbound RNA and the high-FRET population to bound RNA (10). It was noted at the time that although the high-FRET population saturated with increasing RNAP concentrations, the low-FRET population remained, raising the possibility that the low-FRET population represented a second RNAP-bound population that overlapped with the unbound RNA population. Consistent with predictions from structural modelling, we now propose that the high-FRET population represents the vRNA promoter in the pre-initiation state, while the low-FRET population represents another RNAP-bound population in which the 3′ terminus is in the RNAP active site. The RNA may be bound in either a stable pre-initiation or initiation state, and remain stably bound in that conformation, or RNA within a single complex may re-orientate between the two different conformations on a timescale slower than the 1 ms transit time that it takes for fluorescently labelled species to diffuse through the illuminated confocal volume on our microscope.

There are a number of possible explanations for the existence of the pre-initiation state. During an infection, the length of viral RNA not bound by the RNAP is coated with multiple nucleoproteins (NP), forming ribonucleoprotein (RNP) complexes. The pre-initiation state may represent the vRNA conformation found within vRNP complexes packaged into progeny virions, which are not actively replicating. The addition of ApG, mimicking an initial replication product, appears to bias the RNAP complex towards the initiation state, supporting the idea that this state represents an active form. The binding of the 3′ vRNA on the RNAP surface could also represent a ‘waiting’ conformation that the 3′ end adopts after it has been copied and exits from the active site, ensuring that it is located nearby to allow subsequent rounds of transcription to occur with a minimal delay after copying of the gene segment (5). The 3′ end bound on the surface of the RNAP is also compatible with models of replication that invoke the requirement of a second RNAP (13,31,32), as the 3′ vRNA on one RNAP would be available to translocate into the active site of a second, RNA-free RNAP.

We have also shown that the 3′ terminus of the cRNA promoter is likely to form a pre-initiation state. Negative-stain EM has revealed that cRNPs are structurally similar to vRNPs (13), and sequence similarity between the cRNA and vRNA promoters suggests that the cRNA may form a similar structure when bound by the RNAP (8,9). This has been confirmed for the 5′ cRNA (6), which adopts a stem loop structure virtually identical to that of the 5′ vRNA (Supplementary Figure S2). The 3′ cRNA may adopt a pre-initiation state for similar reasons to the vRNA; however, initial replication is likely to differ due to the internal initiation strategy of cRNPs compared to the terminal initiation that occurs on vRNA templates. In our experiments, we observed that addition of RNAP to a final concentration of 100 nM was adequate to ensure that all fluorescent vRNA was bound, as demonstrated by the complete shift in FRET distribution when RNAP was added (for example, in Figure 4A). This was not the case for the cRNA promoter, which required 200 nM of RNAP to fully abolish the unbound FRET population. Previous studies have shown that the purified PB1 subunit of the RNAP bound to the vRNA promoter with higher affinity than to the cRNA promoter (33,34). Consistent with this, we showed that the full-length RNAP has a higher binding affinity for both single-stranded and double-stranded vRNA than cRNA, perhaps reflecting differences in promoter sequences or the different functions of vRNPs and cRNPs.

**Figure 7.**
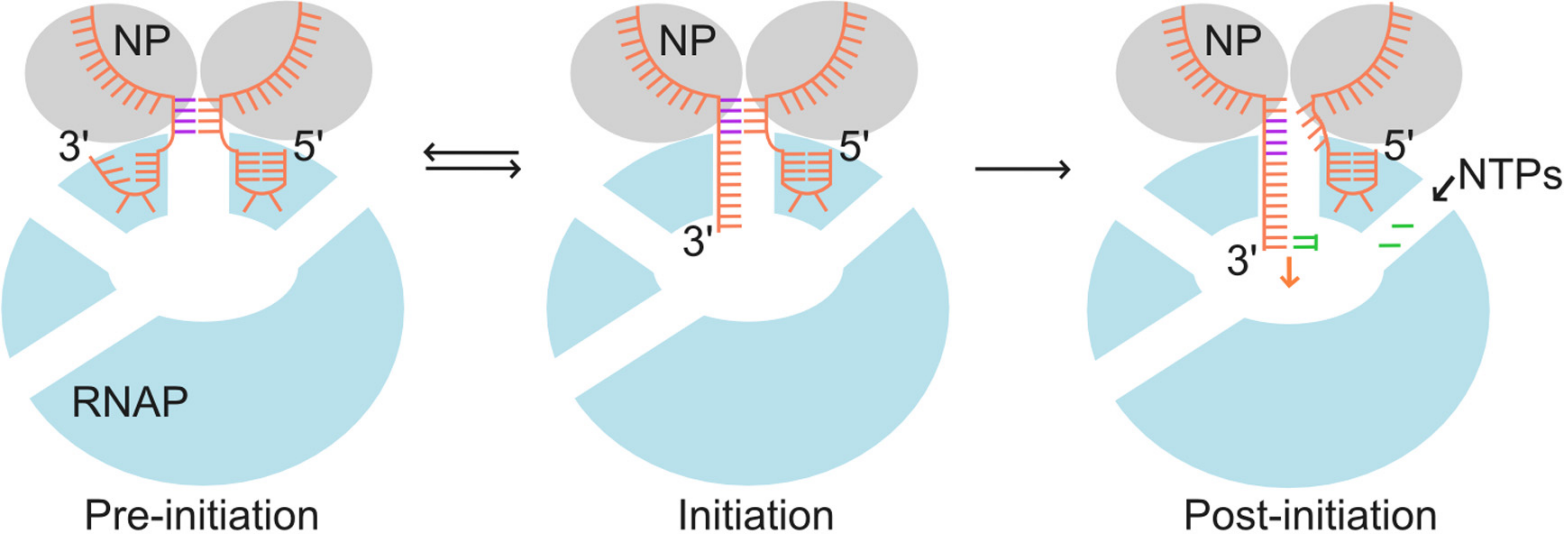
Model of pre-initiation, initiation and post-initiation states. The RNAP is shown in light blue, with the active site depicted as a white oval in the center. Nucleoprotein (NP) are shown in grey. The vRNA promoter is shown in orange, with base-paired residues in the duplex region highlighted in purple and incoming nucleoside triphosphates (NTPs) shown in green. Upon RNAP binding, the vRNA promoter exists in equilibrium between a pre-initiation state in which the 3′ RNA terminus is bound on the RNAP surface, and an initiation state in which the 3′ RNA is bound in the active site. In the presence of NTPs, the 3′ RNA starts to translocate through the active site during RNA synthesis, resulting in destabilization of the duplex region.

Our results support a model in which the RNAP-bound 3′ end of the template vRNA adopts two conformations, consistent with predictions of pre-initiation and initiation states, and whereby translocation of the 3′ vRNA through the active site during initial replication results in destabilisation and processive unwinding of the double-stranded duplex (Figure 7). This model gives new insights into the mechanisms of promoter binding by the influenza RNAP and has implications for the understanding of the replication mechanisms of other negative strand viruses due to the highly conserved nature of their RNAPs.

## SUPPLEMENTARY DATA

Supplementary Data are available at NAR Online.

SUPPLEMENTARY DATA
