# Supplementary material for: Single-molecule FRET reveals the pre-initiation and initiation conformations of influenza virus promoter RNA
Source: Nucleic Acids Res. 2016 Sep 30;44(21):10304–15. doi: 10.1093/nar/gkw884 (PMC5137447; doi:10.1093/nar/gkw884)
Supplement: SUPPLEMENTARY DATA [file supp_44_21_10304__index.html]

Single-molecule FRET reveals the pre-initiation and initiation conformations of influenza virus promoter RNA — SUPPLEMENTARY DATA 

# Single-molecule FRET reveals the pre-initiation and initiation conformations of influenza virus promoter RNA

## SUPPLEMENTARY DATA

- SUPPLEMENTARY DATA
