# Supplementary material for: Single-molecule FRET reveals the pre-initiation and initiation conformations of influenza virus promoter RNA
Source: Nucleic Acids Res. 2016 Sep 30;44(21):10304–15. doi: 10.1093/nar/gkw884 (PMC5137447; doi:10.1093/nar/gkw884)
Supplement: SUPPLEMENTARY DATA [file supp_gkw884_nar-01564-v-2016-File002.pdf]

Supplementary information

Single-molecule FRET reveals the pre-initiation and initiation conformations of the influenza virus promoter RNA

Nicole C. Robb[a], Aartjan J.W. te Velthuis[a,b], Ralph Wieneke[c], Robert Tampé[c], Thorben Cordes[d], Ervin Fodor[b,]* and Achillefs N. Kapanidis[a,]*

[a]Biological Physics Research Group, Clarendon Laboratory, Department of Physics, University of Oxford, Parks Road, Oxford, OX1 3PU, United Kingdom

[b]Sir William Dunn School of Pathology, University of Oxford, South Parks Road, Oxford, OX1 3RE, United Kingdom

[c]Institute of Biochemistry, Biocenter, Goethe University Frankfurt, Max-von-Laue-Strasse 9, 60438 Frankfurt am Main, Germany

[d]Molecular Microscopy Research Group & Single-molecule Biophysics, Zernike Institute for Advanced Materials, University of Groningen, Nijenborgh 4, 9747 AG Groningen, The Netherlands

*To whom correspondence may be addressed: (email) Achillefs.Kapanidis@physics.ox.ac.uk, (phone) +44 (0)1865 272 226, (address) Clarendon Laboratory, Department of Physics, Oxford OX1 3PU, United Kingdom; (email) ervin.fodor@path.ox.ac.uk, (phone) +44 (0)1865 275 580, (address) Sir William Dunn School of Pathology, Oxford OX1 3RE, United Kingdom.

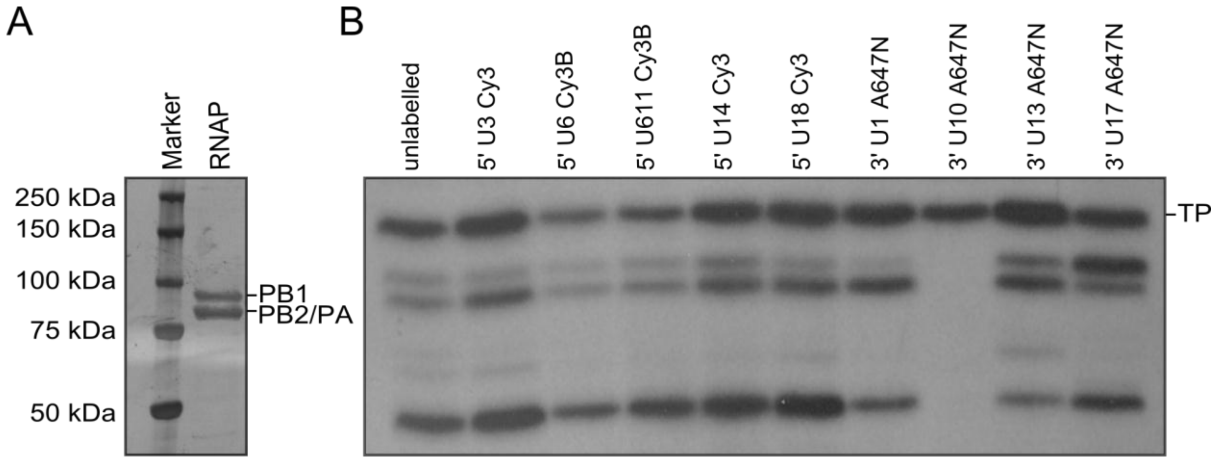

Figure S1. Purified recombinant polymerase is active on fluorescently labelled vRNA promoter in vitro. *(A)* Silver-stained polyacrylamide gel of purified A/NorthernTerritories/60/68 RNAP. Recombinant viral polymerase with a protein-A tag on the PB2 subunit was expressed using the MultiBac system in Sf9 insect cells and affinity purified using IgG-Sepharose followed by size-exclusion chromatography. *(B)* Transcriptional activity of purified RNAP in the presence of 5′ and 3′ vRNA promoter sequences using ApG as a primer (1). The position of the fluorescent dyes in the 5′ or 3′ vRNA is indicated. The full-length 17 nt-long transcription product is indicated (TP); shorter products represent premature termination or degradation products.

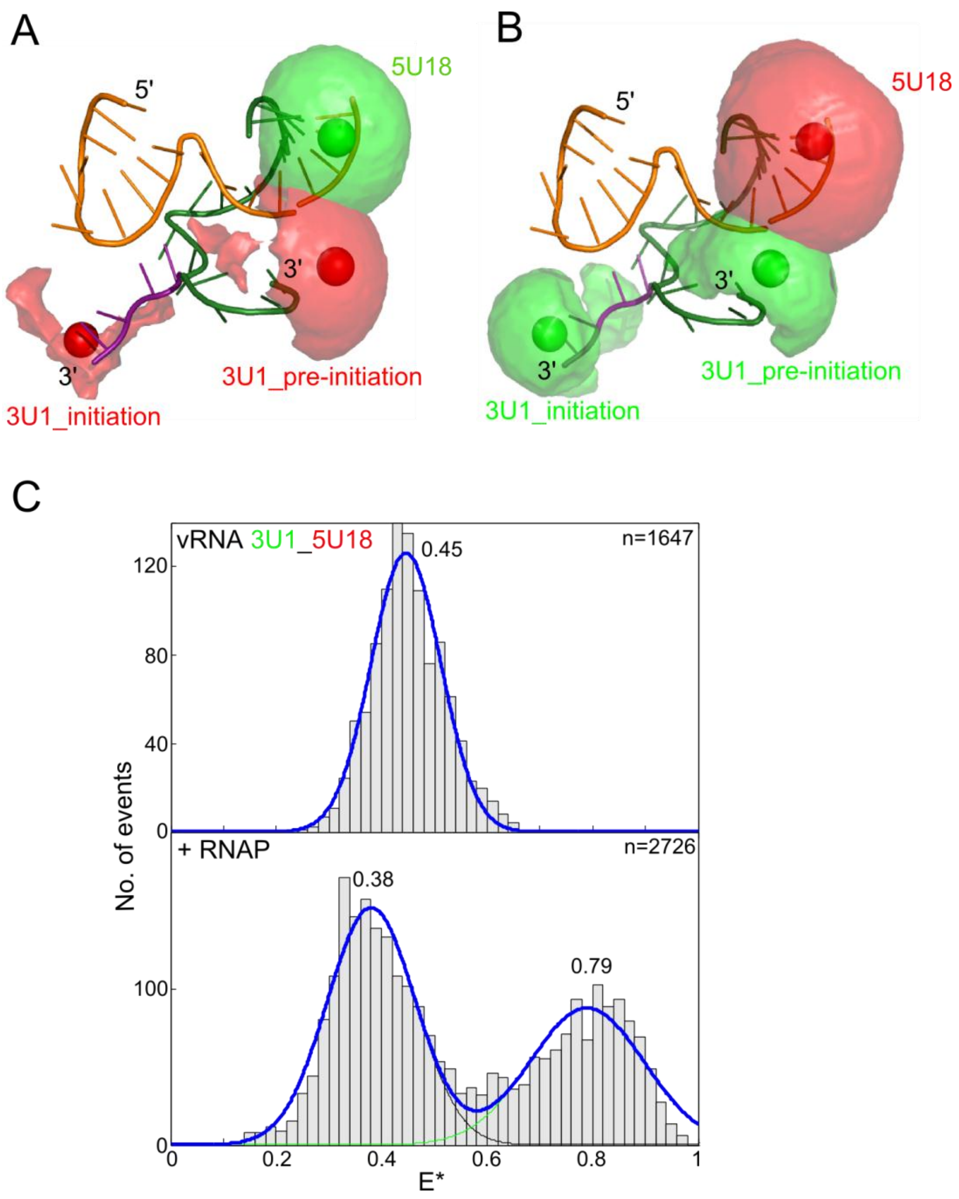

Figure S2. RNAP-bound vRNA promoter with dyes in the opposite orientation also adopts multiple conformations. *(A&B)* The influenza vRNA promoter with accessible volume modelling of the dyes (with the average dye positions depicted as small spheres). The 3' vRNA is dark green or purple, the 5' vRNA is orange, Cy3 dyes are shown in light green and ATTO647N dyes are shown in red. *(C)* Annealed RNAs, corresponding to the 3' (3'-UCGUUUUCGUCCUCAAA) and 5' (5'-AGUAGAAACAAGGAGUUU) ends of vRNA, labelled with donor and acceptor fluorophores at positions 1 on the 3' end and 18 on the 5' end, were analysed alone (top panel), or in the presence of 100 nM RNAP (bottom panel) by single-molecule FRET spectroscopy of diffusing molecules. Ratio E* represents the uncorrected FRET efficiency, n represents the number of molecules and curves were fitted with Gaussian functions to determine the centre of the distributions.

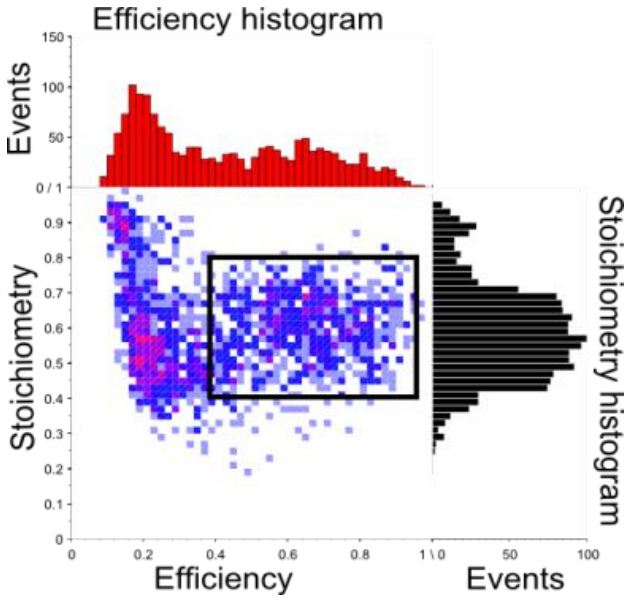

Figure S3. 2D Efficiency-Stoichiometry histogram showing FRET between a fluorescent dye on the RNA and one placed on the RNAP. Cy3-conjugated *tris*NTA was incubated with His_{10} tagged RNAP and a double-stranded RNA labelled with a single ATTO647N dye at position 3U1 before single-molecule FRET spectroscopy of diffusing molecules was carried out. In-situ labelling of the protein led to significant low-FRET populations due to high background from the unbound dye-conjugated *tris*NTA (S~0.9) and coincidence events arising from random collisions of the excess dyes in the confocal volume (S~0.5), however a clear FRET signal (within the black box) could be fitted after appropriate filtering.

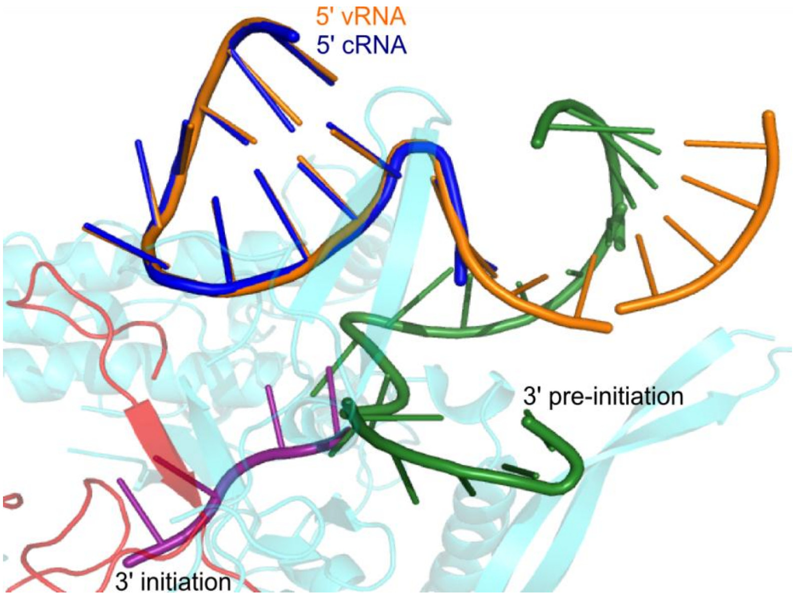

Figure S4. The 5' cRNA adopts a similar stem-loop structure to the 5' vRNA. The RNAP (PDB code 4WSB) is shown in blue, with the active site motifs highlighted in red. The 5' vRNA is orange, 3' vRNA in the pre-initiation state is green and 3' vRNA in the initiation state (from the superposed Norwalk template–primer elongation complex (PDB code 3BSO)) is purple. The 5' cRNA is shown in dark blue.

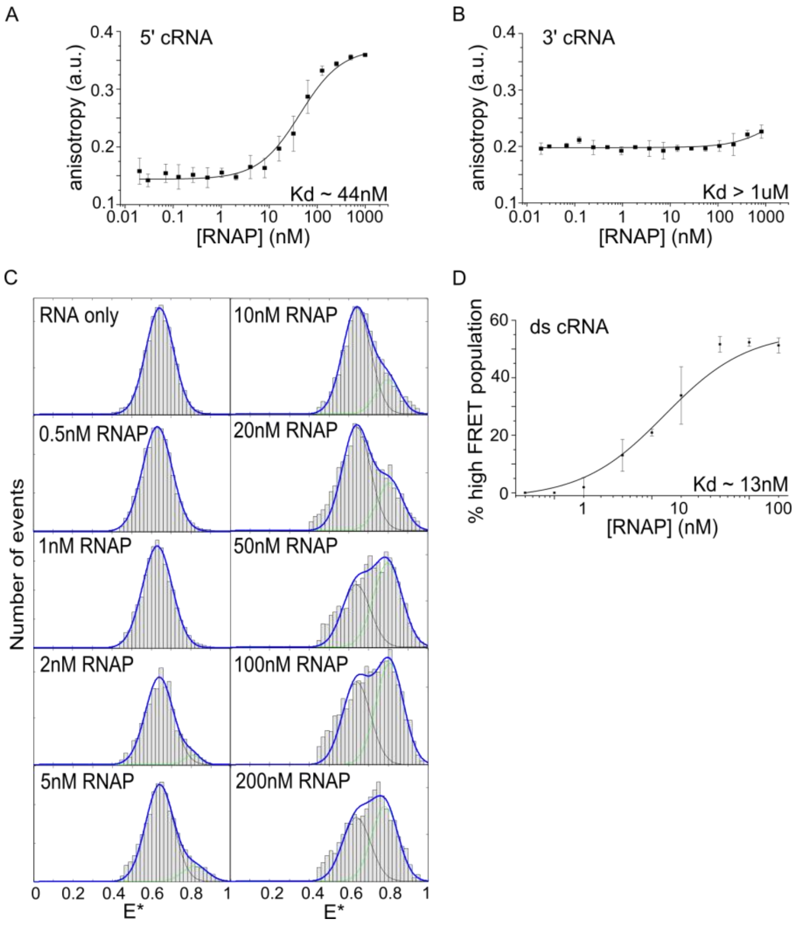

Figure S5. Binding of the viral polymerase to the cRNA promoter. *(A)* Anisotropy curve of the viral polymerase binding to 5' cRNA (5'– AGCGAAAGCAGGAGUUU). Increasing concentrations of polymerase were incubated with 1 nM ATTO647N-labelled RNA for 15 minutes at 28°C and anisotropy was measured in a scanning fluorimeter (Photon Technology International, Birmingham, NJ, USA) with λ_{ex}=640 nm, as described previously(2). Error bars represent the standard error of the mean from three independent experiments. *(B)* Anisotropy curve of the viral polymerase binding to 3' cRNA (5'– UAAACUCCUUGUUUCUACU). *(C)* cRNA promoter was labelled with donor and acceptor fluorophores at position 4 on the 3' end and 17 on the 5' end and incubated with increasing concentrations of polymerase at 28°C for 15 minutes before single-molecule FRET spectroscopy on diffusing molecules was carried out. The lower FRET peaks were fixed at mean E*=0.62, while the higher FRET peaks were fitted freely. *(D)* The area of the high FRET peaks were used to calculate the 'percentage of high-FRET population' at each concentration, plotted against polymerase concentration and fitted with a hyperbolic curve in order to calculate the dissociation constant, as described previously (2). Error bars represent the standard error of the mean from three independent experiments.

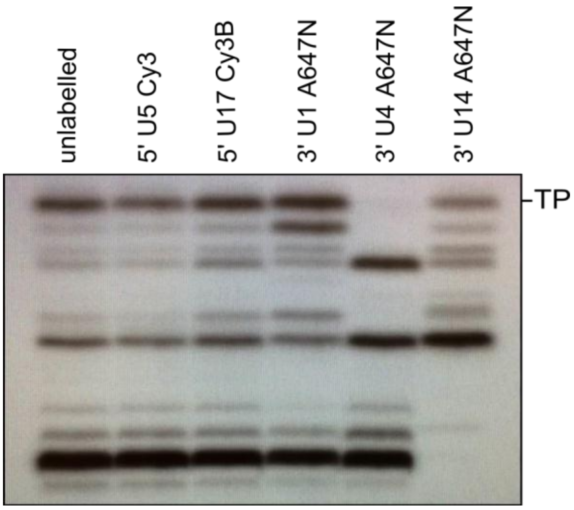

Figure S6. Purified recombinant polymerase is active on fluorescently labelled cRNA promoter in vitro using ApG as a primer. The position of the fluorescent dyes on the 5′ or 3′ cRNA is indicated. The full-length 18 nt-long transcription product is indicated (TP); shorter products represent premature termination or degradation products. We note that a dye at position 4 or 14 on the 3' cRNA results in a different band pattern, however the polymerase is clearly still able to bind and transcribe these templates.

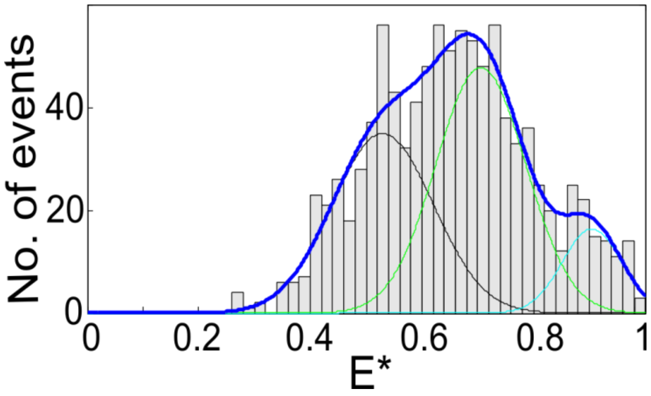

Figure S7. Influenza virus RNAP-bound cRNA promoter adopts multiple conformations. cRNA promoter was labelled with donor and acceptor fluorophores at position 1 on the 3' end and 5 on the 5' end and incubated with 100 nM polymerase before single-molecule FRET spectroscopy on diffusing molecules was carried out. Ratio E* represents the uncorrected FRET efficiency, and curves were fitted with Gaussian functions to determine the centre of the distributions.

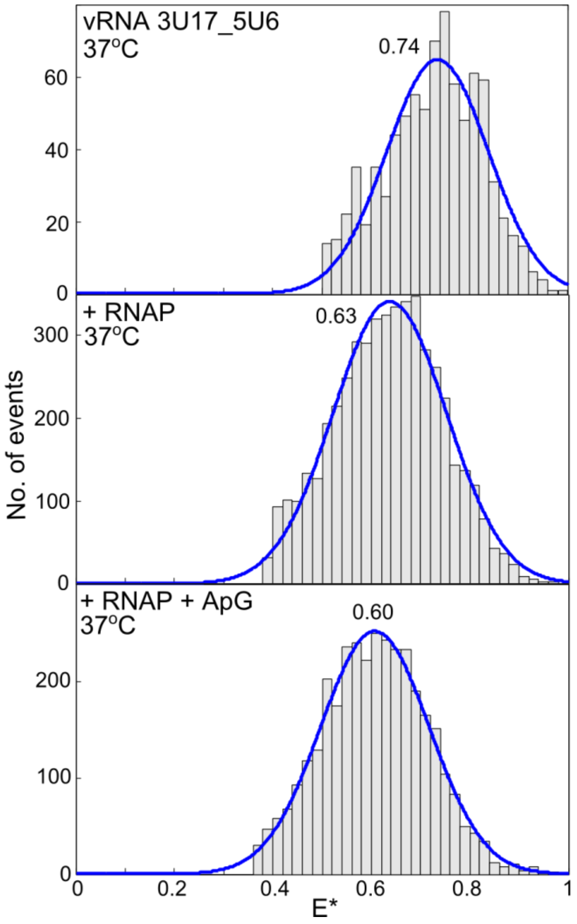

Figure S8. The duplex region of the vRNA promoter is destabilised during initial replication at 37°C. vRNA promoter (5'-AGUAGAAACAAGGAGUUU and 3'-UCGUUUUCGUCCUCAAA) was labelled with donor and acceptor fluorophores at positions 17 on the 3' end and 6 on the 5' end and either analysed alone (top panel), or incubated with 100 nM polymerase (middle panel), or 100 nM polymerase and 500 µM ApG (lower panel) before single-molecule FRET spectroscopy on diffusing molecules was carried out at 37°C.

Supplementary Materials and Methods (not included in the main text)

Fluorescence anisotropy. Increasing concentrations of polymerase were incubated with 1 nM ATTO647N-labelled RNA for 15 minutes at 28°C in 50 mM Tris-HCl (pH 8), 500 mM NaCl, 10 mM $MgCl_2$, 100 µg/µl BSA, 1 mM DTT and 5% glycerol before being placed in a quartz cuvette. Anisotropy was measured in a scanning fluorimeter (Photon Technology International, Birmingham, NJ, USA) with λ_{ex}=640 nm, as described previously (2). The data was plotted in OriginPro8 and a K_d value was extracted by fitting with the following function, where A_o is the initial anisotropy value, A_f is the final anisotropy value, D_t is the RNA concentration and K_d is the binding constant:

$$y=A_o+(((A_f-A_o)*((x+D_t+K_d)-(((x+D_t+K_d)^\wedge 2)-4*x*D_t)^\wedge(0.5)))/(2*D_t))$$

Supplementary References

1. Fodor, E., Crow, M., Mingay, L.J., Deng, T., Sharps, J., Fechter, P. and Brownlee, G.G. (2002) A single amino acid mutation in the PA subunit of the influenza virus RNA polymerase inhibits endonucleolytic cleavage of capped RNAs. *J Virol*, **76**, 8989-9001.
2. Tomescu, A.I., Robb, N.C., Hengrung, N., Fodor, E. and Kapanidis, A.N. (2014) Single-molecule FRET reveals a corkscrew RNA structure for the polymerase-bound influenza virus promoter. *Proc Natl Acad Sci U S A*, **111**, E3335-3342.
